# Vacancy-induced brittle to ductile transition of W-M co-doped Al_3_Ti (M=Si, Ge, Sn and Pb)

**DOI:** 10.1038/s41598-017-14398-6

**Published:** 2017-10-25

**Authors:** Mingke Zhu, Ping Wu, Qiulin Li, Ben Xu

**Affiliations:** 1 School of Materials Science and Engineering, Tsinghua University, Beijing, 100084 P. R. China; 2 Graduate School at Shenzhen, Tsinghua University, Shenzhen, 518055 P. R. China; 3 Singapore University of Technology and Design, 487372 Singapore, Singapore

## Abstract

We investigated the effect of vacancy formation on brittle (D0_22_) to ductile (L1_2_-like) transition in Al_3_Ti using DFT calculations. The well-known pseudogap on the density of states of Al_3_Ti migrates towards its Fermi level from far above, via a W − M co-doping strategy, where M is Si, Ge, Sn or Pb respectively. In particular, by a W − M co-doping the underline electronic structure of the pseudogap approaches an octahedral (L1_2_: t_2g_, e_g_) from the tetragonal (D0_22_: e_g_, b_2g_, a_1g_, b_1g_) crystal field. Our calculations demonstrated that (1) a W-doping is responsible for the close up of the energy gap between a_1g_ and b_1g_ so that they tend to merge into an e_g_ symmetry, and (2) all M-doping lead to a narrower gap between e_g_ and b_2g_ (moving towards a t_2g_ symmetry). Thus, a brittle to ductile transition in Al_3_Ti is possible by adopting this W − M co-doping strategy. We further recommend the use of W-Pb co-doped Al_3_Ti to replace the less anodic Al electrode in Al-battery, due to its improved ductility and high Al diffusivity. Finally this study opens a new field in physics to tailor mechanical properties by manipulating electron energy level(s) towards higher symmetry via vacancy optimization.

## Introduction

**Figure 1:**
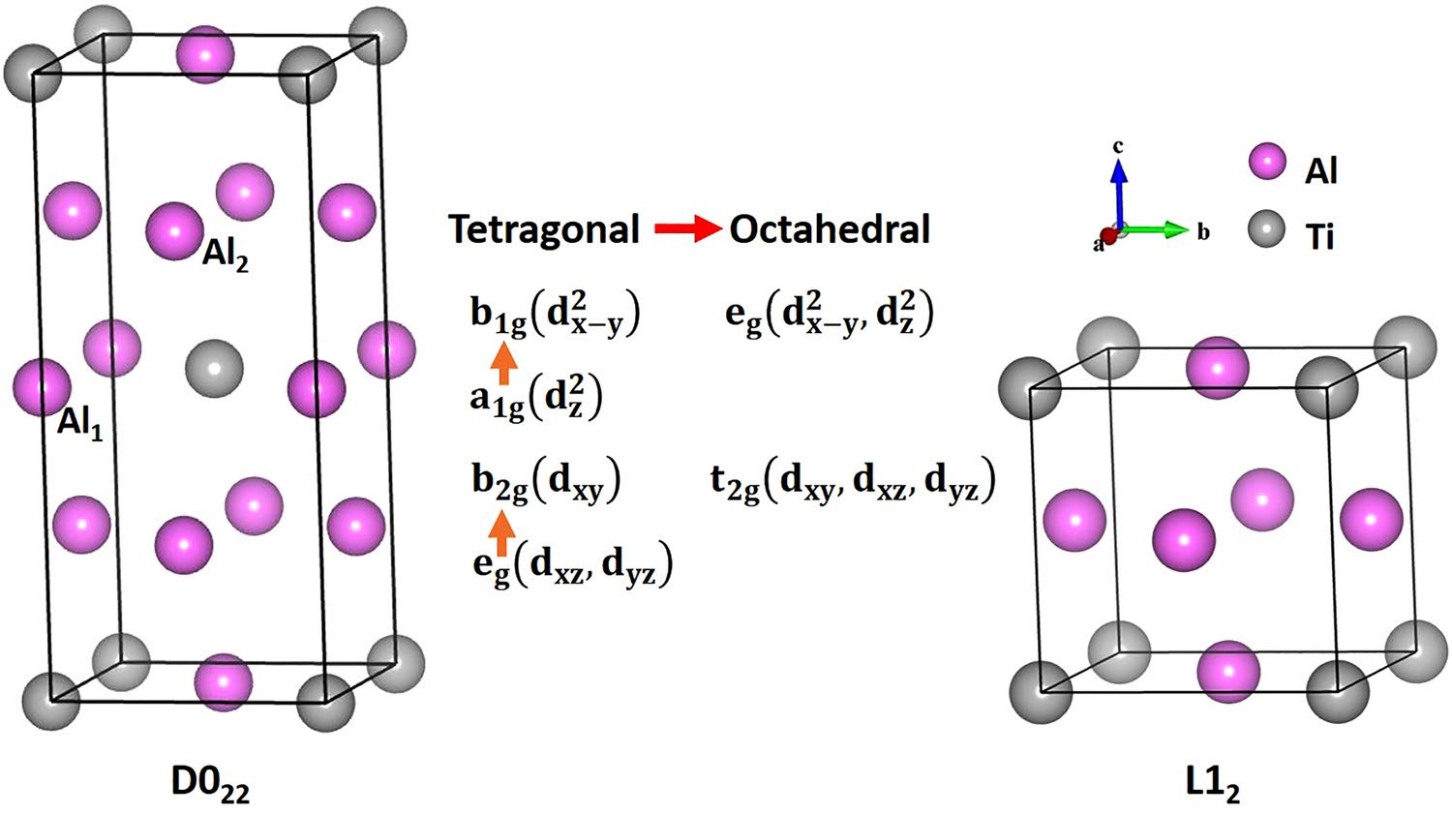
Comparison of the lattice and electronic structures between D0_22_-Al_3_Ti and L1_2_-Al_3_Ti. The schematic diagram between the two structures shows that for the tetragonal D0_22_-Al_3_Ti, the 3*d*-orbital splits into b_1g_ (d^2^_x−y_), a_1g_ (d^2^_z_), b_2g_ (d_xy_) and e_g_ (d_xy_, d_yz_), while for the octahedral L1_2_-Al_3_Ti, the 3*d*-orbital splits into e_g_ (d^2^_x−y_, d^2^_z_) and t_2g_ (d_xy_, d_xz_, d_yz_).

**Figure 2:**
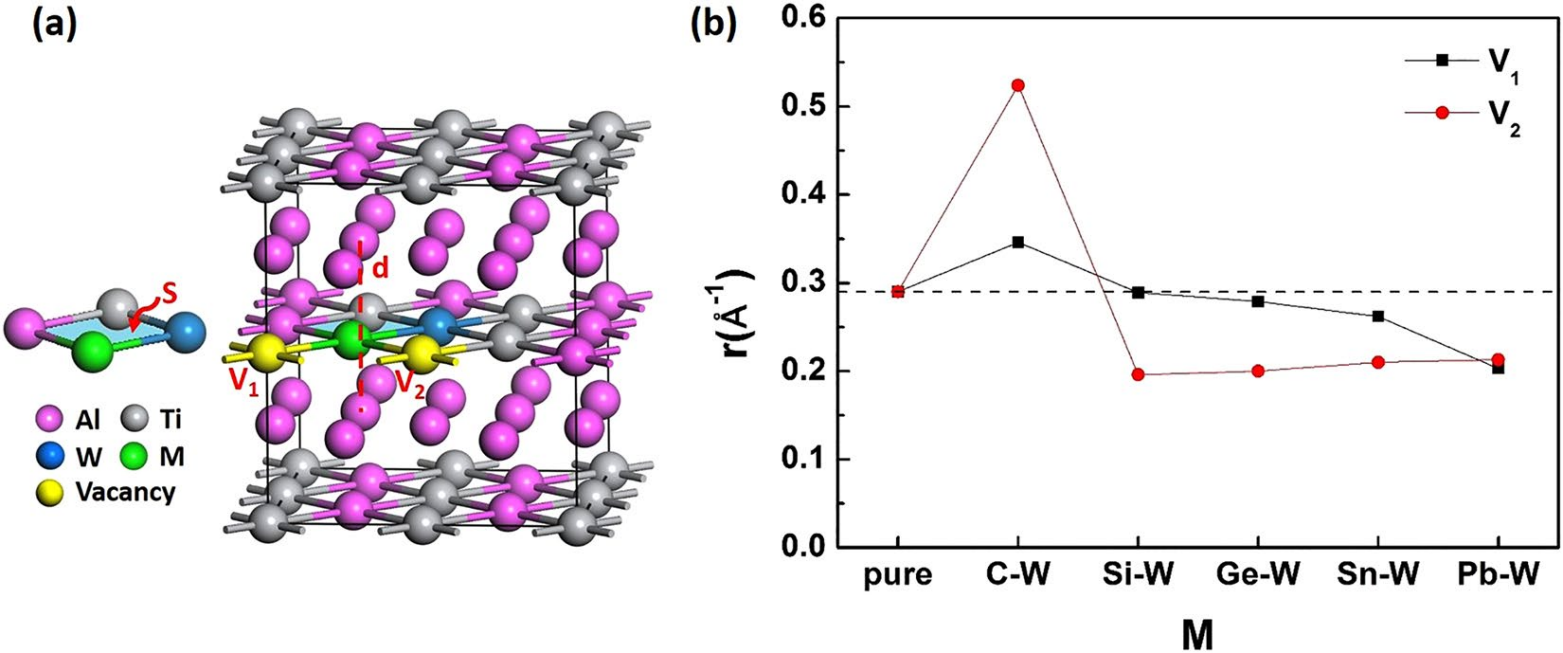
(**a**) Crystal structure of the W − M co-doping Al_3_Ti (Note that the unit cell length (**d**) along the z-axis and the effective unit area (S) are highlighted, where l_x_ is the length between M and Ti and l_y_ is the length between Al and W). (**b**) The calculated ratio *r* (Å^−1^) (*r* = d/S) in pure D0_22_-Al_3_Ti and W − M co-doping systems with an Al vacancy at the V_1_ site.

Brittle to ductile transition is of interest to a wide range of fundamental research and applications^1–6^. In particular, effects of either intrinsic vacancy^7,8^, extra-electron^9^, or dopants^10,12^ on brittle-ductile transition in Nb_5_Si_3_, NiSc, Al_12_W-type and L1_2_-Al_3_Sc are reported. TiAl-based intermetallic compounds are desirable candidates for high temperature structural applications due to many attractive properties. Among the Al-Ti alloys, Al_3_Ti has received particular interests for its high specific strength, elastic moduli^13^, low density (~3.3 g/cm^3^), good thermal conductivity and high melting point (~1400 °C). However, the stable but brittle tetragonal D0_22_-Al_3_Ti is less favored in real applications. Many investigations are conducted aiming to improve the ductility of D0_22_-Al_3_Ti. Hong^12^ calculated the density of states (DOS) of the brittle D0_22_-Al_3_Ti and the ductile L1_2_-Al_3_Ti phases, and proposed a strategy to simultaneously stabilize the ductile L1_2_ and destabilized the brittle D0_22_ phase by ternary alloy additions. He further pointed out that by adding in lower-valence elements, the pseudogap (on DOS) migrates from above to below the Fermi level, thus, diminishing simultaneously the antibonding for the ductile L1_2_ and the bonding states for the brittle D0_22_ phases. But Hong did not take into consideration the formation of either intrinsic or extrinsic defects into his model. On the other hand, Niu^9^ proposed to promote ductile to brittle transitions in Al_12_W-type intermetallic by an extra-electron doping, which is on the opposite direction of the current work. The underline electronic structures of pseudogap in both D0_22_ and L1_2_ Al_3_Ti phases are reported recently by Chen^14^. Crystal field splitting as shown in Fig. 1 is found responsible for the formation of the pseudogaps, i.e., an octahedral crystal field of e_g_ (d^2^_x−y_, d^2^_z_) and t_2g_ (d_xy_, d_xz_, d_yz_) for the ductile L1_2_ phase, and a tetragonal one of b_1g_ (d^2^_x−y_), a_1g_ (d^2^_z_), b_2g_ (d_xy_) and e_g_ (d_xy_, d_yz_) for the brittle D0_22_ phase. It is interesting to notice that the main difference between the octahedral and tetragonal crystal field splitting is the elongation in tetragonal along the z-axis, which relaxes the electron density along the z-axis and moves (1) the d^2^_z_ energy downwards apart from the d^2^_x−y_ and (2) the d_xz_ and d_yz_ lower than the d_xy_ level. An effective unit area as shown in Fig. 2a is defined as S = l_x_ × l_y_, where l_x_ and l_y_ is respectively the shortest atomic distance along the x and y axis. By shrinking the unit cell (or d in Fig. 2a) along the z-axis or expanding in the xy-plane indicated by S, a tetragonal crystal structure may return to and approach an octahedral-like structure. Thus, by reducing the ratio *r* (Å^−1^) (*r* = d/S), a brittle-ductile transition may be facilitated. From first-principles calculations, we test this new strategy to achieve the designed reduction of *r* by adopting a W − M co-doping strategy. Key challenges in this approach are (1) to generate sufficient Al vacancies in D0_22_-Al_3_Ti to make the brittle structure more deformable and (2) to manipulate specific electron energy levels to transfer the low symmetry tetragonal to a high symmetry octahedral-like crystal field.

Moreover, due to the decrease in Gibbs free energy, Al_3_Ti may also be used as the anode material to replace Al in Al-battery. Recently, electronic structure of pure D0_22_-Al_3_Ti^12,14^ and Al diffusion mechanisms of D0_22_-Al_3_Sc^11^ are reported. However, like many brittle intermetallics, short cycling life of a native D0_22_-Al_3_Ti electrode is expected due to the structure damages during the charge and discharge processes. In addition, high Al diffusivity is essential to Al-battery, which requires an easy formation of Al vacancy based on Shi’s^11^ findings that Al vacancies facilitate the Al diffusion in Al_3_Sc. Therefore, the current study on the formation of Al vacancies may provide practical solutions to enhance both the mechanical and electrochemical properties of Al_3_Ti for Al-battery applications.

## Results

### Crystal structure

Crystal structures of both the ductile (L1_2_) and brittle (D0_22_) phases are shown in the Fig. 1. A L1_2_-Al_3_Ti crystallizes in the *Pm* $\bar{3}$ *m* space group, in which the Al atoms are located at the face centers of the cubic lattice and the Ti atoms are located at the vertices. And a D0_22_-Al_3_Ti crystallizes in the *I4/mmm*. The conventional D0_22_ cell contains two Al atoms at the Wyckoff site 2b (defined as Al_1_), four Al atoms at the 4d site (defined as Al_2_) and two Ti atoms at the 2a site. In this study, the lattice parameters are fixed at the values of a = b = 0.3851 nm and c = 0.8611 nm, for c/a = 2.236, which are in satisfactory agreement with other experimental and calculation results^15,16^.

To conduct a systematic study, the site preference of W in D0_22_-Al_3_Ti was investigated first by using a 2 × 2 × 1 supercell including 32 atoms. The first-principles calculations have been performed to calculated the total energies *E*_tot_ for the pure D0_22_-Al_3_Ti supercell and *E*_dope_ for [(Al_23_W)Ti_8_] and [Al_24_(Ti_7_W)] structures. To determine the site preference of W, the substitution energy *E*_sub_ is defined as:

1 $${E}_{{\rm{sub}}}={E}_{{\rm{dope}}}-{E}_{{\rm{tot}}}+{\mu }_{{\rm{Al}}/{\rm{Ti}}}-{\mu }_{{\rm{W}}}$$

where *μ*_i_ (i = Al, Ti and W) is the chemical potential of these atoms in their stable bulk phases. In this study, the stable phases are considered as Ti in *hcp* structure^17^, Al in the *fcc* structure^18^. After occupying Al_1_, Al_2_ and Ti site by a W-atom, the substitution energies of the three structures are −1.089 eV, −0.969 eV and 0.005 eV, respectively. It is clearly seen that the ternary W-atom strongly favors the Al site over the Ti site in the D0_22_-Al_3_Ti. Therefore, we default to substitute W-atom at the Al_1_ site in the following work, as the blue sphere shown in Fig. 2a.

Then the IV-group elements M (M = C, Si, Ge, Sn and Pb) were introduced into the D0_22_-Al_3_Ti/W system and occupied the Ti site to form a W − M cluster, as the green sphere shown in Fig. 2a. The substitution energies of single M-atom doping at the Ti site in D0_22_-Al_3_Ti can be written as:

2 $${E}_{{\rm{sub}}}({\rm{M}})={E}_{{\rm{M}}}-{E}_{{\rm{tot}}}+{\mu }_{{\rm{Ti}}}-{\mu }_{{\rm{M}}}$$

where *E*_*M*_ is the total energy of single M-atom occupying Ti site. While the substitution energies of W − M clusters can be written as:

3 $${E}_{{\rm{sub}}}({\rm{W}}-{\rm{M}})={E}_{{\rm{W}}-{\rm{M}}}-{E}_{{\rm{tot}}}+{\mu }_{{\rm{Al}}}+{\mu }_{{\rm{Ti}}}-{\mu }_{{\rm{W}}}-{\mu }_{{\rm{M}}}$$

where *E*_w−M_ is the total energy of W − M co-doping system. Δ*E* is defined as:

4 $$\begin{array}{rcl}{\rm{\Delta }}E & = & {E}_{{\rm{sub}}}({\rm{M}})-{E}_{{\rm{sub}}}({\rm{W}}-{\rm{M}})\\  & = & {E}_{{\rm{M}}}-{E}_{{\rm{W}}-{\rm{M}}}-{\mu }_{{\rm{Al}}}+{\mu }_{{\rm{W}}}\end{array}$$

The results are showed in Table 1. Δ*E* are positive which means that the co-doping systems have much lower substitution energies than the single doping systems. It indicates that introducing W in pure D0_22_-Al_3_Ti structure will conduce to the substitution of Ti by M.

**Table 1:** The total energies of M doping Al_3_Ti (E_M_) and W − M co-doping Al_3_Ti (E_W−M_), and Δ*E*.

M	C	Si	Ge	Sn	Pb
E_M_(eV)	−160.52	−160.49	−159.85	−157.87	−156.90
E_W−M_(eV)	−169.87	−168.69	−167.85	−165.79	−164.62
Δ*E*	1.945	0.795	0.595	0.515	0.315

### Vacancy formation energy

The crystal model with an Al vacancy were created by removing an individual Al-atom from W − M co-doping supercell. In order to reduce the computation loads, we focus on the first-nearest neighbors, thus, the two possible Al vacancies are at V_1_ and V_2_ sites considering the system symmetry, shown as yellow spheres in Fig. 2a. The stability of the defected structures were studied by vacancy formation energy calculation after the atomic defects are relaxed completely. The formation energy of a neutral aluminum vacancy (hereafter simply referred to as an aluminum vacancy) (${E}_{V}$) is estimated by the following equation (5):

5 $${E}_{{\rm{V}}}({\rm{M}})={E}_{{\rm{def}}}-{E}_{{\rm{W}}-{\rm{M}}}+{\sum }_{i}{n}_{i}{\mu }_{i}$$

where *E*_V_(M) is the vacancy formation energy, *E*_def_ is the total energy of D0_22_-Al_3_Ti/W supercell containing one M-atom and one Al vacancy simultaneously and *E*_W−M_ is the total energy of W − M co-doping supercell. The last term represents the difference in the number of atoms from the W − M co-doping system, where ${n}_{i}$ denotes the number of atoms to be taken from or inserted into the supercell in order to take account of point defect generation. If a corresponding atom is inserted into the supercell, ${n}_{i}$ is negative and if such an atom is taken away from the supercell, ${n}_{i}$ is positive. ${\mu }_{i}$ is the chemical potential of these atoms in their stable bulk phases. The calculated defect formation energies are tabulated in Table 2.

**Table 2:** The vacancy formation energies of W − M co-doping Al_3_Ti when an Al vacancy forms at V_1_ site (${{\rm{E}}}_{{{\rm{V}}}_{1}}$) or V_2_ site (${{\rm{E}}}_{{{\rm{V}}}_{2}}$) under both Al-rich and Ti-rich environment.

	${{\rm{E}}}_{{{\rm{V}}}_{1}}$(eV)		${{\rm{E}}}_{{{\rm{V}}}_{2}}$(eV)	
	Al-rich	Ti-rich	Al-rich	Ti-rich
W-C	2.00	1.47	−0.08	−0.61
W-Si	1.15	0.62	−0.07	−0.59
W-Ge	0.95	0.43	−0.14	−0.67
W-Sn	0.42	−0.11	−0.41	−0.94
W-Pb	−0.59	−1.11	−0.59	−1.05

From Table 2, it can be easily observed that the vacancy formation energies of Al at V_1_ site are higher than that at V_2_ site. For a W − M co-doping system, when the Al vacancy occurs at V_2_ site, the vacancy formation energies are negative under both Al-rich environment and Ti-rich environment, which indicates V_2_ defects can be formed spontaneously during the fabrication of the alloy. In addition, both V_1_ and V_2_ defects are spontaneously formed by a W-Pb co-doping under either Al-rich or Ti-rich environment. The results attribute to the fact that the W − M co-doping cluster plays a vital role in the formation of Al vacancies.

### Electronic states of defected structure

**Figure 3:**
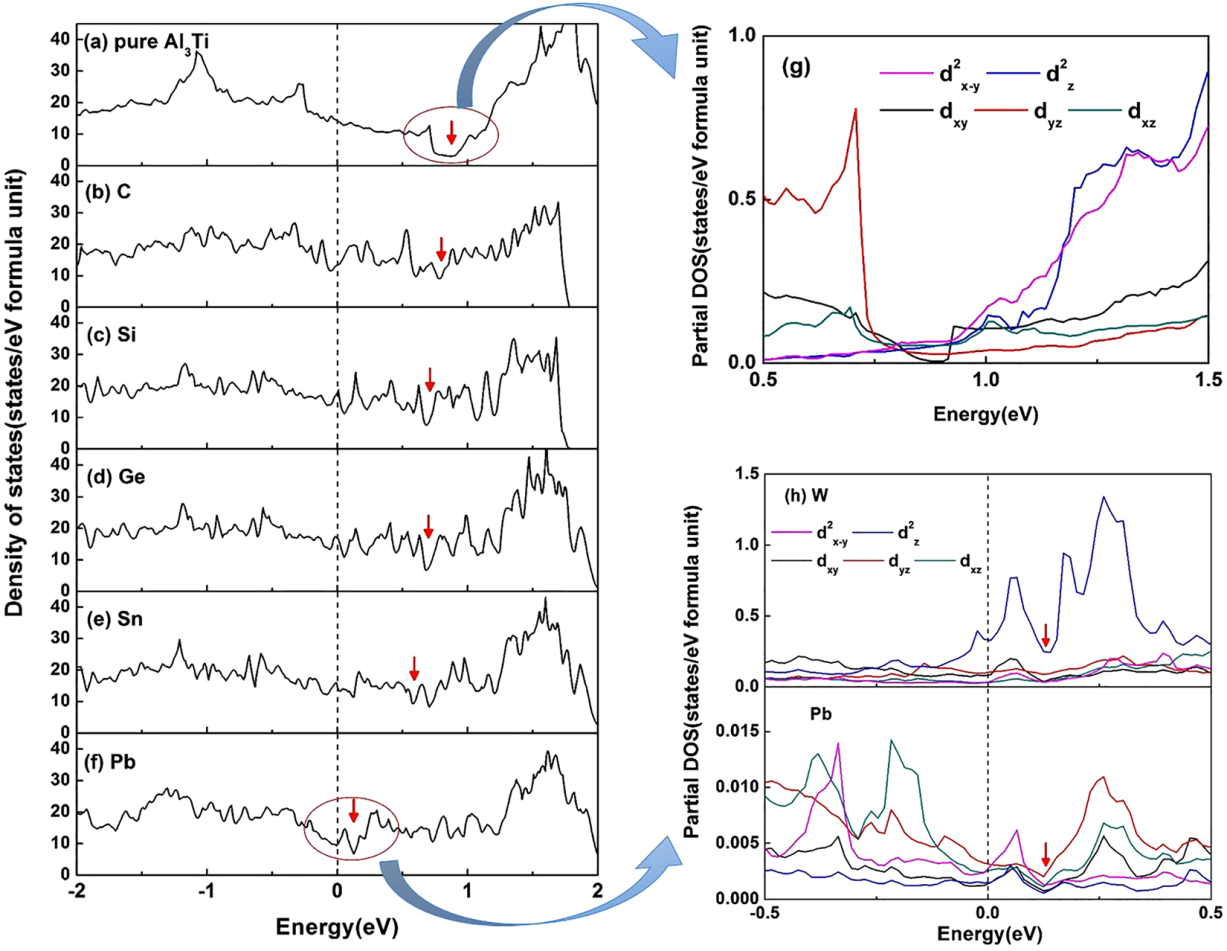
The calculated (DOS) of (**a**) pure D0_22_-Al_3_Ti, (**b**–**f**) W − M co-doping Al_3_Ti with an Al vacancy at V_1_ site, W = C, Si, Ge, Sn and Pb, respectively. The calculated partial DOS of (**g**) pure D0_22_-Al_3_Ti, (**h**) W-Pb co-doping Al_3_Ti (Note only 3d orbitals are presented here). All arrows indicate the psuedogap position.

The calculated DOS are given in Fig. 3a for the pure D0_22_-Al_3_Ti, and in Fig. 3b–f for W-C, W-Si, W-Ge, W-Sn and W-Pb co-doping Al_3_Ti with an Al vacancy at the V_1_ site, respectively. Δ*n* is introduced to indicate the valley of psuedogap. The value of Δ*n* is the energy of the lowest position on the calculated DOS curve. Thus, the positive value means the psuedogap is higher than the Fermi level. The result is shown in Table 3. A clear pseudogap is observed in the D0_22_-Al_3_Ti (circled part in Fig. 3a), which indicates the strong bonding-antibonding separation. The result shows a strong hybridization existing in the D0_22_ structure as well as a strong directionality in bonding. Therefore, it is difficult to form the slip system in the tetragonal D0_22_ structure and leads to brittleness. The partial DOS of D0_22_-Al_3_Ti around the pseudogap was investigated, shown in Fig. 3g. From the edges of the gap, the splitting 3d orbitals could be observed clearly, thus, b_1g_ (d^2^_x−y_) energy is higher than a_1g_ (d^2^_z_) on the right edge, while b_2g_ (d_xy_) energy is higher than e_g_ (d_xy_, d_yz_) on the left edge, which appears a typical tetragonal crystal field.

**Table 3:** The position of the valley of psuedogap.

	pure Al_3_Ti	C	Si	Ge	Sn	Pb
Δ*n*(eV)	0.876	0.798	0.684	0.678	0.587	0.125

From Fig. 3b–f, by adding different M elements and forming a W − M co-doping cluster with an Al vacancy at V_1_ simultaneously, the pseudogap migrates from far above towards the Fermi level, indicated by the red arrows. The results show that there are less bonding states which may favor a D0_22_ to L1_2_-like transition. To carry out a more in-depth and detailed study, partial DOS crossing the pseudogap of the W-Pb co-doping system was calculated and shown in Fig. 3h. Contributions from W and Pb to bonding electrons were investigated separately. On the right edge of the pseudogap, it is observed that W-atom contributes a lot to form strong hybridization between the d^2^_z_ and d^2^_x−y_ levels. Similarly on the left edge of the pseudogap, the Pb-atom has a strong influence on rising the d_xz_ energy and d_yz_ energy towards the d_xy_ level (or a strong hybridization among these 3d orbitals). Therefore, the vacancy-induced 3d-orbital-splitting tend to facilitate a ductile L1_2_-like structure, thus, e_g_ (d^2^_x−y_, d^2^_z_) and t_2g_ (d_xy_, d_xz_, d_yz_).

To obtain the brittle to ductile transition, the tetragonal D0_22_ structure is expected to transform into an octahedral-like structure, which could be realized by either a shrinking along z-axis or an expanding on the xy-plane or both. By representing the ratio *r* of z-axis d to the xy-plane S, the change in structures are quantified, as shown in Fig. 2b. Taking pure D0_22_-Al_3_Ti as the standard, it can be concluded that *r* decreased with the formation of Al vacancy at V_1_ site, which indicates that the tetragonal crystal field tends to transform into an octahedral-like crystal field. As a result, the stable phase change from D0_22_ to L1_2_–like ductile structures. When an Al vacancy forms at the V_2_ site, S remains nearly a constant except for the W-C co-doping. The larger S in the W-C co-doping system is due to the small size of C. Among all M elements in Table 4, C is the only dopant whose size is smaller than that of Al (0.39 Å for Al^3+^). More details will be outlined in Session 3 below.

**Table 4:** Ionic Shannon radius^24^ (R_M_) and the electronegativity (EN)^25^.

	C	Si	Ge	Sn	Pb	Al	Ti	W
R_M_(Å)	0.16	0.4	0.53	0.69	0.78	0.39	0.42	0.66
EN	2.50	1.74	2.02	1.72	1.55	1.47	1.32	1.40

## Discussion

In order to enable a brittle to ductile transition, we proposed and validated a W − M co-doping mechanism to (1) generate sufficient Al vacancies in D0_22_-Al_3_Ti, and (2) simultaneously to manipulate specific electron energy levels to approach the high symmetry octahedral-like electronic structures. In particular, an equation for the lattice energy of W − M co-dopants is derived based on the E_W−M_ (eV) given in Table 1:

6 $${E}_{{\rm{W}}-{\rm{M}}}=-170.3+9.44{R}_{{\rm{M}}}^{2}$$

where *R*_M_ is the ionic radius of M.

The calculated E_W−M_ based on equation (6) is −170.1, −168.7, −167.6, −165.8, −164.6 eV for M = C, Si, Ge, Sn and Pb respectively, which is very close to the DFT calculations, thus, −169.9, −168.7, −167.9, −165.8, −164.6 eV. Therefore, E_W−M_ is in proportional to the cross-session of an M-ion (${{\rm{R}}}_{{\rm{M}}}^{2}$), or E_W−M_ is 2-dimensional size (or xy-plane) dependent only. This is a good indicator that a W − M co-doping may only manipulate the xy-plane while leaving out the z-direction untouched.

Similarly, an equation for the formation energy of V_2_-W − M co-dopants is derived based on the ${{\rm{E}}}_{{{\rm{V}}}_{2}}$ (eV) data given in Table 2:

7 $${\rm{For}}\,\text{Al}-\text{rich}:{E}_{{{\rm{V}}}_{2}}=-3.9{({R}_{{\rm{M}}}-0.39)}^{2}$$

8 $${\rm{F}}{\rm{o}}{\rm{r}}\,\text{Ti}-\text{rich}:{E}_{{{\rm{V}}}_{2}}=-0.55-3.9{({R}_{{\rm{M}}}-0.39)}^{2}$$

Both equations (7) and (8) reasonably reproduce DFT calculations shown in Table 2. We derived V_2_-W − M equations only since they are stable (or having negative formation energy) for all the M elements. Like equation (6), both equations (7) and (8) are in proportional to the cross-session changes of a substitutional M-ion and an Al vacancy (*R*_M_−0.39)^2^. Once again, a V_2_-W − M co-doping may only manipulate the xy-plane while leaving out the z-direction untouched. This 2-D manipulation function of W − M co-doping is the basis that enables a brittle (D0_22_) to ductile (L1_2_-like) transition, which can be applied not only for Al_3_Ti but all intermetallics in general.

Finally, we have systematically investigated a series of W − M co-doping D0_22_-Al_3_Ti (M = C, Si, Ge, Sn and Pb) intermetallics using first-principles calculation method. The site preference of W in pure D0_22_-Al_3_Ti was first studied, it shows W (a *d* element) has a clear preference to substitute Al_1_ (a *sp* element) site due to the strong crystal field. Then, we confirmed the [(Al_23_W)Ti_8_] system is conductive to the subsequent doping of M-atom. Meanwhile, a M substitution of Ti reduces the stability of [(Al_23_W)Ti_8_], which might benefit the intercalation and deintercalation of Al-ion during charge-discharge cycling in rechargeable Al-battery. The two possible Al vacancies were also investigated. In comparison to the vacancy formation energies of Li-ion in Li_3_N^19^ (−0.14 ~ 0.52 eV), the Al vacancies in W − M co-doped Al_3_Ti have much lower formation energies, therefore, high Al diffusivity is expected.

The DOS of W − M co-doping Al_3_Ti with an Al vacancy at the V_1_ site were investigated. The results show the pseudogap migrates towards the Fermi level from far above, indicating a tendency to transform into ductile L1_2_-like structure. By analyzing the partial DOS around the pseudogap, we found that W and Pb have almost independent contributions to the transition, thus, W mainly influents d^2^_x−y_ and d^2^_z_ while Pb have a strong effect on d_xy_, d_xz_ and d_yz_. It shows the crystal splitting effect on the 3d orbitals plays a decisive role not only on the formation but also the transformation of pseudogap. Therefore, this study contributes to the formation of a new field in physics to design mechanical properties from electronic structures via vacancy optimization.

## Methods

Calculations were carried out within the framework of density functional theory (DFT)^20^, using the projector-augmented wave (PAW) method^21^ and the Perdew-Burke-Ernserhof (PBE)^22^ for the exchange-correlation energy functional, via the Vienna ab initio Simulation Package (VASP)^23^. We first calculated the equilibrium lattice parameters of the Al_3_Ti using plane-wave cutoff energy of 340 eV and a 7 × 7 × 7 k-point mesh in the Monkhorst-Pack scheme^13^ by using the 2 × 2 × 1 supercell including 32 atoms. In all calculations, self-consistency was achieved with a tolerance in the total energy of 0.01 meV, and the atom were relaxed until the forces were less than 0.01 eV/Å. The crystal structures were fully optimized by independently modifying lattice parameters and internal atomic coordinates.

### Data availability statement

All data generated or analyzed during this study are included in this published article.
